# Supplementary material for: Integrating genomic structural equation modeling and experimental validation to unravel the genetic basis of male genital lichen sclerosus
Source: Front Immunol. 2026 Jul 16;17:1857945. doi: 10.3389/fimmu.2026.1857945 (PMC13435250; doi:10.3389/fimmu.2026.1857945)
Supplement: Supplementary file 6 [file SupplementaryFile5.zip › result/fuma_PSYCH_factor_sCCA2_focus.chr16.chr16.49007926.52035823.pop1.pdf]

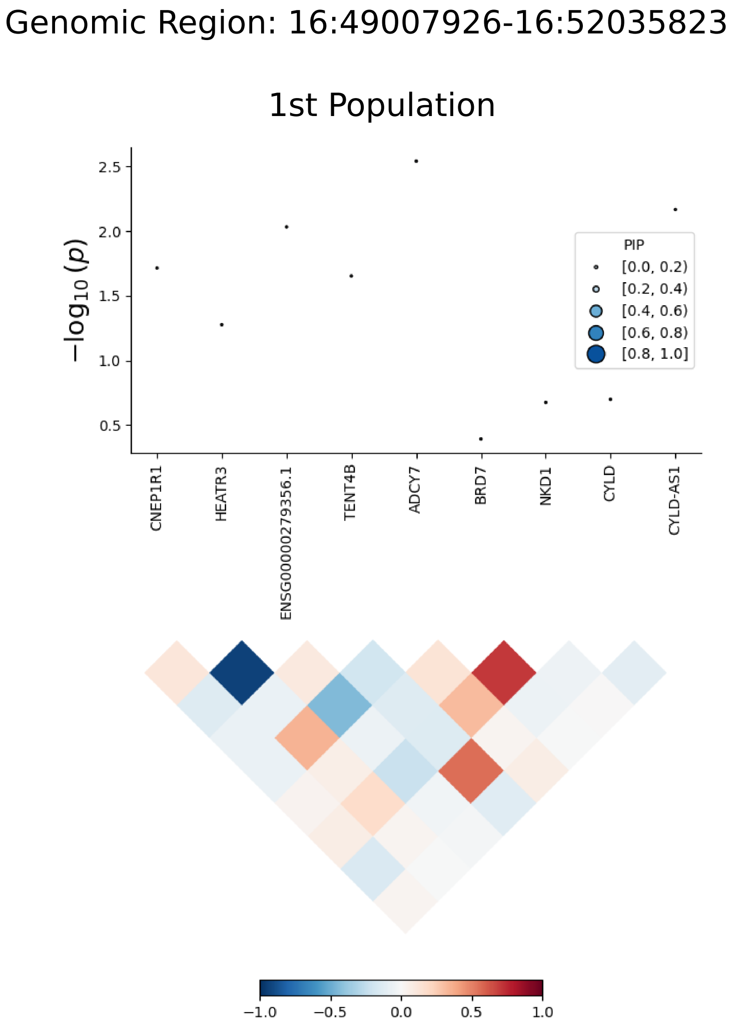
Genomic Region: 16:49007926-16:52035823
1st Population
−log10(p)
2.5
2.0
1.5
1.0
0.5
PIP
[0.0, 0.2)
[0.2, 0.4)
[0.4, 0.6)
[0.6, 0.8)
[0.8, 1.0]
CNEP1R1
HEATR3
ENSG00000279356.1
TENT4B
ADCY7
BRD7
NKD1
CYLD
CYLD-AS1
−1.0
−0.5
0.0
0.5
1.0
